# Lusophony Pentecostal Churches in Berlin: Religious Identities Between Integration and Transatlantic Boundaries

**DOI:** 10.1007/s41603-021-00154-2

**Published:** 2021-12-07

**Authors:** Stefan van der Hoek

**Affiliations:** Faculty of Theology, Friedrich Schiller University, Fürstengraben 6, 07743 Jena, Germany

**Keywords:** Religion, Pentecostalism, Lusophony, Migration, Network

## Abstract

Although migration is a constant in human history, current trajectories have new quantitative and qualitative features with religious implications, which are addressed in this article. What is new and paradoxical about what is commonly referred to as globalization is the diffuse nature of worldwide migration and the mobility of people, ideas, and goods. This article therefore explores how members of Brazilian Pentecostal congregations in Berlin use specific functions and patterns of interpretation communicated or generated by discourses of the churches to cope with the lack of social capital in a new social and cultural environment and how their interpretations and orientations shape everyday actions. This article is an attempt to capture the ambiguous role of religious resources in the process of migration and social integration as well as the actions of community members. Although a growing body of literature explores the influence of migrant organizations on its members, new church actors of Christian migrants in Germany are rarely considered as drivers of religious pluralization. Therefore, this article reflects on the different functions of Brazilian Pentecostal congregations for the integration of Lusophone Pentecostal migrants in Berlin. In order to identify the functions of Pentecostal organizations, a theoretical framework is determined and related to the statements of the interview partners and to findings from observations. To answer the research question, this article draws on field research conducted between November 2019 and June 2020. The empirical analysis uses data from four narrative interviews and over 40 participant observations in four different transnational congregations belonging to a Brazilian Pentecostal network of churches. The results show that individual religiosity and belonging to a particular religious group not only provide social relationships and a network of solidarity for individuals, but also reinterpret the social exclusion and marginalization of migrants.

## Introduction

A first preliminary remark concerns the landscape of European religion, in which a diverse spiritual geography can be observed and the settlement of new immigrants represents a significant driver of new religious pluralization (Knott 2017). This pluralization affects the appearance of Christianity in the different European countries and promotes developments which have been described by other researchers as the “De-Europeanization of Christianity” (Andreyeva 2017). An increasing number of Christian organizations are molding and reshaping the identity of Christianity from below in many European countries (Pasura and Erdal 2016). The different groups that can be held responsible for these processes of pluralization are extremely diverse and have different cultural, linguistic, and religious backgrounds. Also, the conditions of religious pluralization are diverse among European states and require close examination regarding their historical, demographic, and political structures. The German political system can be characterized as an inclusive model of religious governance in which recognized mainline churches are treated as institutional partners of the state and are entitled to certain rights, resources, and privileges (Kortmann 2020). Given the present situation, this explicitly political role of the mainline churches challenges the situation of minor religious organizations on different levels, and the unequal balance of power and visible presence of new religious actors have in some cases caused new conflicts (Paas and Bartholomä 2020). Especially the city of Berlin has been a notable place for proponents of the rational-choice theory to demonstrate medial, clerical, and governmental stigmatization and discrimination against new religious actors, such as US Pentecostalism (Stark and Finke 2000). However, Pentecostalism and Pentecostal organizations have a long history of stigmatization in Germany (Eisenlöffel 2006; Simpson 2011). Even though Pentecostalism has been present in Germany since the beginning of the twentieth century, it remains a marginalized group until today (Areani 2013). However, Pentecostal associations in Germany are currently benefiting from the influx of Christian migrants and the Pentecostal movement is becoming stronger in numbers due to global migration flows. While in 1992, there were only 13 migration churches registered in the largest Pentecostal umbrella organization named *Bund freier Pfingstkirchen (BfP)*; in the year 2019, there were already 325 Pentecostal migrant churches registered.^1^ The correlation between migration and the local diversification of Christianity is in line with other analyses that examined the changing constellation of Christianity in other European countries. Especially local concentrations of Pentecostalism in Europe are often associated with a strong presence of migrants from the Global South (Jenkins 2008).

This brings me to another essential preliminary remark regarding the global trajectories of migration from the Global South to Europe and to Germany. Among the OECD Member states, Germany has become one of the popular destination countries for immigration worldwide (Bergseng et al 2019). While Brazilian migration to Germany has increased significantly and Brazilian culture is blooming in the urban centers of Germany, the religious dimension of Brazilian and Lusophony diaspora in Germany is still neglected in international research (Oro 2004; Vaz Feijo 2015). In Germany, academic and public discourses on migration and religious pluralization often refer to the “non-Christian” religious presence as drivers of religious pluralization and diversification and neglect that most of the immigrants during the past few decades have come from countries dominated by Christianity, such as Italy, Spain, Poland, or Portugal (REMID 2017). The French Huguenots are only one among other prominent Christian groups who have enriched the Christian landscape in Germany through migration. Across Germany, Russian Brethren congregations, Nigerian Pentecostal churches, Spanish and Polish Catholic congregations, and Korean Presbyterians can be found contributing to the diversification of the German religious landscape. The non-denominational Christian association *Migrationskirchen-in-Berlin e.V*. estimates that there were roughly 250 different congregations of migrants in the year 2016 in Berlin, of which 200 were classified as Pentecostal churches.^2^ Although the relevant scientific community has no clear conventions yet regarding the terms used to describe churches planted by migrants, I will use the terminology of a»migrant church« to indicate a Christian community established to minister to a diasporic group of people who share a common religious, ethnic, and linguistic background. It is important to note that migrant churches can be very different from the churches of the host society and also their countries of origin. The communities often find themselves in complex and ambiguous relationships between global and local realities, past and present, profane and sacred, continuities, and discontinuities. The self-understanding of a religious majority or minority may automatically affect the actions of the church and its members. Also, marginalization, discrimination, and precarity can impact migrants’ well-being, status, and social relationships on many different levels and affect the social coherence in a migrant church. Transcontinental and transatlantic migration to the European Union can have an impact on the recognition of certain skills and specific qualifications, which are often either revoked or reduced by the local labor market. In addition, housing issues and residential mobility may perpetuate instability. Unfamiliar cultural and social norms and the emotional toll of familial and social separation may add up to seemingly insurmountable challenges for migrants. Religious affiliations and interpretations can therefore be used by migrants as a form of empowerment and vocalized in a religious community such as a migrant church. Migrant churches try to respond in a special way to the needs of their congregants and to meet them through spiritual offerings. Especially Pentecostalism is in this sense often seen as a catalyst of social and cultural changes. In this understanding, da Silva Moreira (2014) identifies a threefold function of Pentecostal churches, which will be reflected in this article against the background of the Brazilian Pentecostal churches in Berlin.

A first function identified by da Silva Moreria is to detach members from their traditional affiliations by weakening their ties to the same. A second function is to push the process of individualization through personal empowerment. Traditionally interpreted as the gifts of the Holy Spirit, which are used to increase the social capital of individuals in the community and to give each member the opportunity to contribute through spiritually interpreted talents. As a third function, da Silva Moreira addresses the communal offering for globally disembedded individuals in diaspora and migration situations to re-embed themselves through their social networks as well as strategies to rearticulate identity. As da Silva Moreia points out, Brazilian Pentecostal churches play an important role in the globalized context as agents of individualization, promoting critical reflection on traditional culture and generating positive liberating forces (ibid). These functions will be discussed further in this article in light of the findings of my fieldwork.

## Methodology

This article investigates the functions described by da Silva Moreira among Pentecostal churches of Brazilian Migrants in Berlin. In doing so, it brings together empirical explorations and theoretical concepts of cultural sociology and sociology of religion. Based on two methodological approaches, this article presents four Brazilian Pentecostal churches in Berlin. Transnational mobility became a research topic at the beginning of the twentieth century and was primarily focused on analyzing how social order can be ensured despite migration and pluralization (Diaz-Bone 2010). However, with the increasing influence of the Chicago School and other researchers, the focus switched to the individuals, their experiences, and induvial transformation processes caused by migration (Park 1928). In this sense, the focus of this article is to identify and to reflect on the question how Brazilian Pentecostal churches can serve the three functions described by da Silva Moreira (2014). Drawing on more than 40 participant observations and four semi-structured narrative interviews, this article brings to light the actual diversity of the Brazilian Pentecostal landscape in Berlin and their functional potential. The data for this article were acquired during a master’s program in *Practical Research in Social Work* at the Alice Salomon University in Berlin. The Master’s thesis was based on the research question how Lusophony migrant churches in Berlin can be understood as places for social integration and serve as a migrant association addressing the social needs of Brazilian migrants in Berlin. The interviews were conducted and observations were taken in Berlin between November 2019 and June 2020. However, the research was overshadowed by the global spread of the COVID-19 pandemic, which slowed down the process of acquisition. Possibly, more comprehensive data would have been obtained under other circumstances. Throughout the observations, I placed a strong analytical focus on prayers, languages, communications, and other forms of social interactions before, during, and after the church services. Over the course of my participatory observations, I engaged in many informal talks and discussions with an array of members, deacons, assistants, and pastors of the different churches. All names of interlocutors, insofar as they are mentioned in this article, are pseudonyms.

## Brazilian Migration to Germany

From the 1960s and 1970s onwards, Europe began to receive significant numbers of immigrants from Brazil (Stelzig-Willutzki 2012). This first major wave consisted mainly of opposition politicians and intellectuals who were forced to leave the country temporarily after the *Coup D’Etat* in 1964. The Brazilians exiled to Europe and Germany during this early period were highly qualified and fluent in several languages. In many cases, they returned to Brazil after a gradual (re-)democratization had begun in the 1980s (ibid). Another factor contributing to the mobility from Brazil to Germany was the new mass tourism of West-German citizens to Brazil in the late 1980s, which led migration researchers to assume that this phenomenon is a possible reason for the increasing number of young female Brazilian immigrants who fell in love with male German tourists and therefore moved to Germany (ibid). However, this perspective on transatlantic migration might only be a Eurocentric explanation, while other researchers propose that the increasing emigration—especially during this period—was due to alarming social inequalities, rampant crime, and violence (Vásquez and Rocha 2013). Still, and until today, the proportion of women among Brazilian migrants in Germany remains unusually high. According to the Brazilian Ministry of foreign affairs, 53.8% of the 491,243 Brazilians abroad are female.^3^ However, the number of naturally born Brazilian women in Germany is disproportionately high at 65.1% (BAMF 2020) and reached even 77.8% (BAMF 2008) in the year 2007. This demographic detail might be relevant to the structures in Brazilian Pentecostal churches which are highly feminized, as international researchers have frequently pointed out (Chesnut 1997; Mafra 2002; Oosterbaan 2017).

Even though Brazil experienced a period of economic growth and political stability during the 2000s, the number of emigrants increased rapidly until today. Whereas 6122 people immigrated from Brazil to Germany in the year 2000, the number rose steadily up to 13,599 new immigrants in 2019 (BAMF 2020). The different reasons for transatlantic migration may vary tremendously. The reasons and motives for the massive increase in emigration are ambiguous and still suggest a variety of possible interpretations. Margolis (2013) noticed the remarkable boost of Brazilian immigration to Europe and puts it in the context of the events of the terroristic attack of 9–11, after which the US government put in place stricter requirements for immigration.

The immigration of Brazilians to Europe and especially to Germany, however, can only be attributed to multiple causal inferences. It occurs primarily in the larger urban centers in Germany, such as Hamburg, Munich, Frankfurt, and Berlin, and the presence of Brazilian culture in those urban centers is particularly visible in the public sphere and can be evaluated as highly appreciated among the German population. Various Brazilian restaurants and grocery stores can be found there. The demand for Brazilian cultural is also expressed in Samba Schools and Capoeira or Jiujitsu courses. Brazilian snacks and performing artists, folk festivals and even a Candomblé temple can be found in contemporary Berlin (Brito de 2020). With the Brazilian migration, however, also Brazilian churches came to Berlin, which so far have not been the subject of research contributions.

## The Landscape of Brazilian and Lusophony Pentecostalism in Berlin

In the section that follows, the Brazilian Pentecostal congregations and the interviews with the congregants will be presented and described in relation to the research question. I would like to mention that there were more Lusophony groups and Brazilian churches to be identified during my fieldworks, which could have been included into this research as well but did not agree to be included or are lacking a comparable organizational structure. The church *Deus é Amor* has declined to be included in the research for no apparent reason. Another church which could have been included into the research is the Brazilian church *Bola de Neve*, but it has so far only organized itself as a Facebook group and only occasionally holds informal meetings in parks or private homes. Due to its informality and low level of organization, the group has not been included in this research. A Baptist church in Berlin-Schöneberg has also integrated an Angolan Worship group, in which Pentecostal elements might be found. However, this group is also difficult to compare with the other congregations and was therefore not considered in the research. Moreover, an Angolan New Apostolic Church can be found in present-day Berlin, which in a broader understanding of the definition can be considered part of the Lusophony Pentecostal landscape in Berlin. However, the classification of the New Apostolic Church as Pentecostalism has proven to be difficult on various levels (Haustein 2021). A final significant reason for the selection of four churches was the feasibility of this research. During the period of research, I spent my weekends attending Friday evening services at the *Igreja Universal do Reino de Deus*, Saturday services at *Lagoinha*, Sunday morning services at the *Igreja Universal do Reino de Deus* at 8 a.m., the *Assembléia de Deus* at 10 a.m., and the *Videira* Church at 4 p.m. Observing another church would probably have been outside the logistical possibilities of a single researcher. With this in mind, this article will only depict a spectrum of a larger Lusophony landscape in Berlin.

## *Assembléia* de Deus

The *Assembléia de Deus (AD)* has a comparably long history in Brazil and has already been mentioned and discussed on several occasions (Mafra 2002; Chesnut 1997; Oosterbaan 2017). Also, its international spread has attracted attention in the scientific community, and the *AD* is a prominent example for Brazilian Pentecostalism of the first wave among international researchers (Freston 1995; Anderson 2005; Meyers 2018). The numbers of *AD* churches in Germany are difficult to evaluate. Across Germany, several churches with this name can be identified. A Portuguese-speaking website^4^ about Lusophony churches in Germany lists 14 different congregations under this name but does not include the one in Berlin and can therefore be understood as only an incomplete list.

The observations show that the *AD* in Berlin can be described as an independent congregation which holds only loose ties to other *AD* congregations. This form of organization has also been observed by researchers, who describe the *AD* as a compilation of local and independent communities (Mafra 2002) or as a complex web of geographically entangled networks of mother-churches and dependent churches (Freston 1995), respectively.

Like Kitani characterized the *AD* churches in Japan, the congregation in Berlin manages its affairs independently from other congregations (Kitani 2016). In contrast to Japan however, the congregation in Berlin neither gathers in sophisticated ecclesiastical edifices and has uniformed choirs, nor is its members active in church meetings on weekdays after work. The congregation in Berlin is located in the district of Reinickendorf and rents space in a former store. Although the church is situated in a residential area with a high population of migrants and other visible religious institutions, the *AD* cannot be identified as such from the outside. The darkened store windows remain closed by a curtain. The community in Berlin is therefore different from those in Brazil. While Oosterbaan describes the *AD* in Brazil as a visible community that tries to dominate the public space (2017), the community in Berlin remains shielded from the outside.

Although other *AD-*congregations in Germany, e.g., in the cities of Hagen and Munich, joined the Pentecostal umbrella organization (*BfP*) in Germany, the *AD* in Berlin has no contact to other churches in Berlin or Germany.

The room in which the congregation holds its weekly meetings is very cramped. The church would like to move to more adequate rooms but is having difficulties finding appropriate locations.

Every Sunday before the church service starts, the congregation holds an extensive *Escola Dominical* to instruct its members in the Holy Scripture and to elaborate a common understanding of the Bible. The number of biblical texts covered is high. In each of these Sunday Schools, six to sixteen different Bible passages are counted and covered in detail. In some cases, keywords are also be looked up in their original ancient Greek or Hebrew interpretation. This density of text passages is a special feature and indicates the high authority of the Holy Scripture. The *Escola Dominical* is sober and determined by a scripture-based discussion. Often, memories from Brazil are processed into everyday situations and discussed among the participants. Negative examples from other congregations in Brazil and their theologies are reflected with striking frequency, and they supposedly help the congregation to sharpen its own profile and self-image.

While approximately up to 10 people gather for the *Escola Dominical*, up to 40 people including children come to the Sunday church service, which takes place immediately after the *Escola Dominical*. The small room fills up quickly and strollers block the paths between the rows of chairs. A band with drums, trombone, keyboard, and clarinet huddles rapidly together and starts playing the music.

The musical material that is played during the church services is traditional and old fashioned. The songs often come from the *Harpa Cristã*, a song collection by the *AD* from 1922, which contains songs like “*How Great Thou Art*” by Carl Boberg and “*A Mighty Fortress Is Our God*” by Martin Luther in their respective Portuguese interpretations. The community sings the songs with pleasure and enthusiasm. Songs, as well as oral communication, take place exclusively in Portuguese. Only the Bible text is projected on the wall in Portuguese as well as German. During the church services, the members share their personal experiences with God or pray for family members and friends abroad; songs are sung, children are blessed, and the collection of the tithe takes place.

The age of the audience is more diverse than in the other Brazilian churches in Berlin because of the elderly people who also join the *AD* church service. Nevertheless, the average age can be described as young, which is mainly due to the large number of children in the community. The memories and shared knowledge from Brazil play an important role during and after the church service; many of the visitors come together to enjoy Brazilian food or to chat in Portuguese. Although many of the worshipers are originally from Brazil and the topics of the worship services relate almost exclusively to the Brazilian social context, there is also a group of Portuguese speaking African adherents who visit the community regularly but do only interact in the social activities with the Brazilians seldomly. Also, the formal offices in the church are held exclusively by Brazilian’s members.

Within the congregation, organizational power appears to be decentralized and is designated to the numerous deacons and church assistants. The pastor, who serves the congregation, is from Rio de Janeiro. He is around 60 years old and has previously worked as a music teacher in Brazil. He plays the keyboard and likes to integrate his musical talent into the church services. He has no certified theological training and his German language skills are “poor,” as he mentions himself in one of his sermons. After a personal conversion experience, according to the pastor’s testimony during of the sermons, he had received a divine call to be a missionary abroad and initially spent several years in Italy before coming to Berlin. As a pastor, he enjoys high recognition and authority inside the church, and many adherents and visitors consult him on relevant issues. Interestingly, he is also a consultant on issues regarding migration, integration, and social participation for the congregants. However, the pastor cannot live up to these high expectations, which was a particular complaint in an interview with one of the church members.

Because of the limited financial resources of the congregation, the pastor works fulltime and cannot provide as much time as the members of the church need. The interview in the church was conducted with Alessandro. Alessandro used to be an engineer and lawyer in Brazil, and most recently worked as an entrepreneur. As a senior, he married a younger woman and became a father of two children only a few years ago. His wife works for an international company, and the family moved to Berlin two years ago. The family’s income is good, and Alessandro feels comfortable in Germany. He enjoys the city but finds it difficult to socialize, so the *AD* is a suitable place for him to get in touch with interesting people, as he says. Challenges he is facing concern his son’s parenting and schooling, since his own language proficiency in German is not sufficient to have conversations with his son’s teachers.

Alessandro must adjust to a completely different life due to migration. Previously in Brazil, he worked as an entrepreneur and enjoyed recognition. In Berlin, however, he works only as a househusband, which can be a challenge, but Alessandro manages it without difficulty and has settled well into his new role. Compared to other interviewees, Alessandro does not resort to a spiritual pattern of interpretation to cope with the situation but is happy that his wife is a successful manager in a well-known international company.

Although the *AD*’s image of family is seen as very conservative in Germany and the sermons emphasize a clear hierarchy and social differences between men and women, Alessandro does not seem to accept this interpretation for himself.

Further reflection on the research question and the congregation, however, gives rise to an interesting observation. As the observations have shown, the *AD* is solidified in its traditions and structures. The members seem to redefine these traditions for themselves and use room for interpretation for themselves.

Participating in the worship seems to be an important routine for its members; it is important to be there, to engage in discussions and singings. However, the congregation does not step outward and does not try to dominate the public space or to reach out to people across the neighborhood, as in Brazil. Preservation and conservation in a diasporic situation seem to be key terms to describe the *AD* in Berlin. The *AD* provides a network of solidarity and spiritual support, which is not to be underestimated, but at the first glance, it offers only little to no bridges to the German host society, and neither does it seem to fulfill any of the functions which were proposed by da Silva Moreira.

### Videira

The literature about the *Videira* resonates strongly with the international evangelical literature and receives attention by authors such as Timothy D. White (2012), Johnson and Clark (2011), Randy Clark (2017), and Joel Comiskey (2020), who highlight the *Videira* church as a prolific global example for healing experiences, successful church growth, and church planting. The *Videira* is still a relatively young community, and research about its background and its history is still scarce in the academic literature. In Brazil, the church was originally founded in Goiâna (Nobre 2003). Derived from previous congregations and movements, the *Videira* emerged and became a larger network within Brazil and quickly started to expand internationally (Rodrigues 2007). Although the leadership’s actual expansion target were African and other Latin American countries, the church was also able to successfully spread within the international Lusophone migrant milieu on different continents (Mareels 2015). In Europe, the *Videira* is represented in eight countries.

The church in Brussels has already been explored in other research papers by Elisabeth Mareels (2015) and helps as an academic reference. In an interview with Mareels, Pastor Aluizo, founder and current lead-pastor of the *Videira* church, reported that *Videira* was able to attract a larger number of young Germans to volunteer in social projects for the church in Brazil. It is worth mentioning that, at the time of the interview, military service had just been abolished in Germany and young Germans were taking advantage of the opportunities offered by the new *Bundesfreiwilligendienstgesetz* (engl. Federal Volunteer Service Law) to engage in a social project after schooling. Young people used the opportunity and supported social and charitable projects in Germany as well as abroad.

According to Aluizo, especially during this time, the *Videira* benefited from approximately 100 volunteers from Germany who were involved in the projects of the *Videira* church in Brazil (ibid.), although he did not mention any correlation with the changing laws regarding military service. From the extraordinary high number of 100 young Germans, 12 volunteers decided to stay in the church in Brazil and three of them were trained as pastors who could then be deployed in Europe, according to Aluizo in the Interview (Mareels 2015).

In Germany, currently four congregations belong to the network of the *Videira*. The churches are located in Frankfurt, Karslruhe, Hamburg, and Berlin. The history of *Videira* in Berlin started in 2012. The church sees itself as a vineyard and the single congregation as a vine with a multitude of grapes, which symbolize small groups within the communities. The small groups are characteristic for the *Videira* and represent an important aspect in the life of the congregants, which also distinguishes it from other Brazilian churches in Berlin. Unlike the other churches, *Videira* does not mainly focus on its spiritual activities during the Sunday service or on the weekly gatherings under the supervision of a pastor, but it focuses rather on the interpersonal relationships of the individual believers of the community. Although the pastor enjoys a high level of authority within the community and the Sunday services play an important role for the congregants, interpersonal fellowship seems to be the primary spiritual focus.

Believers receive plenty of spiritual support and pastoral guidance from their friends. They pray for each other in cafés, restaurants, subway stations, or parks, read the Bible together, or share thoughts about God and prophecies. Therefore, small groups entail important spiritual and social functions for their members that must not be underestimated. The small, family-like community meet every week in private homes and imitates and, in some way, replaces the lost networks of friends and family in Brazil.

Social interactions dominate the time of the meetings of the small groups: eating, gaming, and other social activities are very much appreciated among participants. The meetings in the living rooms begin with emotional worship, music videos are played on television, and the participants have the opportunity either just to listen to the songs or to pray for each other.

For the individuals of the community, the organization of small groups can have further important functions: In case of conflicts between members or the discomfort of a member within a group, the groupings can be changed without a member having to leave the church. The individual groups are very eager to increase in size and to get new members. During my visits, I regularly received invitations to join other groups. Different congregants approached me after the first church service I visited and invited me on a certain day of the week to eat pizza together or play games. Every small group has its own leader or leader team, usually a married couple, who make their premises available for the weekly meetings. Usually songs are sung, prayers are spoken, prophecies are given, and potato chips and soft drinks are consumed all at the same time. The *Videira* in Berlin has managed to expand rapidly, and it has difficulties finding suitable rooms for its weekly Sunday services. At the time of the fieldwork, the parish met every Sunday in the basement of an old brick building owned by the Salvation Army. The basement had to be rearranged for every event, and it took a lot of effort to prepare and install the technology and set up the chairs. At the beginning of 2021, however, the *Videira* was able to obtain its own premises in Berlin.

The Sunday service brings people from other small groups together, to sing together, to pray, to celebrate the Lord’s Supper, to announce events, and to listen to the pastor’s sermon. The congregation also watches short video clips during the services, which show pictures, music, and other impressions of *Videira* congregations outside of Germany and outside of Brazil in order to raise awareness of the congregation’s global growth. The songs in the worship service vary between German, English, and Portuguese songs and have a strong pop-rock-like character. A worship-leader and a band consisting of keyboard, drums, several guitars, and singers encourages the audience to sing along and clap hands. The atmosphere in the services can be described as very emotional. The pastor is often in tears as he speaks about the visions for the future of the church and repeatedly calls out the name of Jesus in a trembling voice. For the believers, this is interpreted as an indication that the Pastor is filled with the Holy Spirit at that moment.

The sermon unfolds its spiritual power only through the social interaction with the congregants. The pastor often calls out keywords and phrases in the sermon and incites the members to repeat them to each other. If the Pastor says, for example, “*God is calling you!*,” he immediately encourages the audience to say to their immediate neighbors, *“God is calling you!”* as well, irrespective of whether or not they know each other. The community’s image of God is consistently and radically positive. Everything good comes from God, while everything negative consequently comes from the devil. In some testimonies, even small gifts, attention, and sweets that believers receive from others are immediately interpreted as a divine gift and shared in testimonies during the services.

The collection of tithes plays an important role in the worship service as well. In envelopes, the tithe is brought to the front and put on a table by the believers who are strictly instructed by a moderator or the pastor not to give the money out of a tradition or sense of duty, but always out of a desire to do so for God.

The audience is comparatively young and is mainly middle-class migrants. Most visitors to the church can be described as cosmopolitans and have a highly individualized lifestyle. They are often well integrated structurally, have jobs in international companies, and communicate in English or Portuguese in their daily lives. Only a few of the visitors speak German fluently.

In one interview, Anna, a young woman from northern Brazil, told me about her conversion story from Catholicism to Pentecostalism and her experiences of migrating to Germany. The beginning of Anna’s spiritual search is the question of the purpose of life. Anna described her life in Brazil as a normal one, as that of many other young women in Brazil. She went out regularly, attended festivities, and had many friendships and relationships with young men.

Her first contact with Pentecostalism came through her mother, who started attending a Pentecostal church, but Anna did not yet understand the contents that were being talked about.

After Anna found affiliation with a Pentecostal church in Brazil, a process began, as she describes it, in which she began to reflect on her life. She began to reevaluate her own family history and to look at biographical obstacles in her or her mother’s life at different educational stages.

In the Pentecostal church in Brazil, Anna found approval of her desire to emigrate to Germany, which she interpreted as a divine sign to fulfill a mission in Germany. She confidently moved to Germany without money or any form of security, except some support by an aunt living in Berlin, and seemed confident that God had a mission for her to accomplish in Berlin and would therefore take care for her. For Anna, the church services in Berlin play a special role, as she explained, but her real hope is grounded in the personal prayers during the small group meetings or with her friends from the church. She interprets the personal prayer as an effective way to prepare for administrative obstacles, since she is receiving regular renewals of her tourist visa in Germany. As simple as it may seem, Anna finds it a proverbial miracle to have God’s people around her, praying for her and sharing her language. Especially in the process of her visa renewal, personal prayer and encouragement through prophecy within a small group is a powerful resource for her. Anna describes the extension of her visa and the opportunity to start a job as a waitress as a blessing and confirmation from God that she is in the right place for her destiny. This awareness helps Anna to acknowledge and reinterpret the devaluation of her professional training as a physical therapist. As she presents it, Anna would never work as a waitress in Brazil, but because God opened doors for her in Germany and gave her an honorable occupation, she feels especially valued in her new work. Anna understands her personal relationship with God as an ongoing development that began when she got to know God personally and received the Holy Spirit eight years ago. Since then, she has been personally changed and talks to God incessantly. In other cases, God sends people, such as friends and co-workers, through whom God speaks to her, according to Anna’s interpretation. This interpretation was also prevalent among the other congregants of *Videir*a during my visits and also corresponds to the content of the sermons on Sunday. Throughout the interview and observations, it became visible on different occasion that the church sees itself as a distinct group that must accomplish a divine mission to evangelize the people of Berlin and are part of a worldwide revival that starts from Brazil.

The *Videira* creates a common identity to which everyone can contribute through his or her individual abilities and gifts. Such an interpretation undoubtedly has a noticeable impact on subjects’ self-image of not seeing themselves as part of a marginalized group, as can be seen in the example of Anna. She interprets her physical environment in a spiritual sense, which became particularly visible during the interview when she talked about the German host society and how it carries a spiritual burden from its past of the Shoah and the two World Wars. In her opinion, this burden could also be related to the cold weather which makes the soil especially hard and barren for mission. Anna describes the people of the German society around her as zombies who have died spiritually and need to be revived.

She experiences difficulties, especially in the financial area and in questions regarding her visa extension. However, Anna receives spiritual and material support from her family and church members to overcome these obstacles. The church provides a network of solidarity for her, and Anna is thankful for the relationships and the support she receives. In the community, she meets like-minded people who confirm the idea that she and the group are on a divine mission. Anna finds social relationships and trust with people she has not known for very long but are an important resource for her after arriving in a new country.

In this sense, the *Videira* can indeed be understood as a catalyst and fulfills many of the functions described by da Silva Moreira. In this instance, the example of the *Videira* shows that Brazilian Pentecostal churches are not only a catalyst for Brazilian people and their religious concerns. The *Videira* knows how to appeal to an international audience that even US evangelical missionaries align themselves with, as evangelical literature depicts.

In the church, immigrants, who often lack long-term relationships, gather with others and create social cohesion based on similar worldviews and spiritual missions. The theology and concepts of God used by the members of the church create a strong positive way of thinking.

### Lagoinha

The *Lagoinha Baptist Church* was mentioned by several researchers in recent years (Miller and Morgan 2019) and is especially known for its worship bands, which gave it national and even international popularity (Gearin and Cavnar 2016). Compared to the *Lagoinha* churches in Brazil, the musical part of the services in Berlin is rather marginal. Often the music is produced by a guitarist or it is played on a computer. The *Lagoinha* has its origins in the district of the same name in the Brazilian city of Belo Horizonte. Even though the congregation calls itself a Baptist church, it has incorporated numerous Pentecostal elements into its worship practices. Speaking in tongues is practiced during prayer, and proclamations for healing and the narrative of spiritual warfare are commonly parts of the church services. The *Lagoinha* started expanding relatively late after its establishment in the 1950s. Since 1972, the main pastor of the congregation is the televangelist Márcio Valadão, who fundamentally reformed the congregation and turned the small traditional Baptist church successfully into a modern transnational mega-church with several hundred thousand members and congregations throughout Brazil as well as in the USA, Australia, Africa, and Europe.

However, the community’s national expansion began in the late 1990s as a coincidental result of a fundraising project. The congregation successfully produced and sold worship music in Brazil and therefore received first-time national attention. Because of the generated attention, other churches joined the *Lagoinha* in Belo Horizonte, and new churches were formed. According to the church, there are currently 500 congregations named and affiliated with *Lagoinha*, 200 of which are abroad.^5^ In Brazil, professional singers are often hired to sing in the Worship-bands of the *Lagoinha* churches. Sermons with musical accompaniment add emotion, and church services are often held in darkened rooms, which also creates a certain kind of concert atmosphere. Just as the rather haphazard global growth of the *Lagoinha* church had begun, it continued in Germany. A group of local congregants who were unsatisfied with the local *AD* church and thus sought to establish a new church in Berlin became part of the global *Lagoinha* branch by opening a *Lagoinha* church in Berlin. The *Lagoinha* church in Berlin was founded 2018 by a Bolivian man and an elderly woman originally from Manaus. Both have no theological training and are pastors because the congregation recognizes them as such due to their charismatic talent, charitable qualities, and social engagement.

Compared to the *Lagoinha* churches in Brazil, the *Lagoinha* in Berlin distinguishes itself in various ways. The worship style in Germany is more conservative and reserved. At the time of the observations, the core of the congregation consisted of no more than 10 people. Tourists from Brazil, friends, and family members would occasionally visit the services as well. In the case of the *Lagoinha* in Berlin, the church rents its service hall from another German Baptist church in Berlin-Charlottenburg. However, the required rent is extremely high for the small congregation, and especially during the COVID-19 pandemic, the congregation had financial difficulties paying the rent to conduct worship services in the church. Alternatively, meetings were held in parks and public places.

Therefore, tithing plays a big role in the community, as it does in the other Brazilian communities in Berlin. Especially abroad, new start-up churches have difficulties generating the necessary funds for purchases and renting. Services take place on Saturday afternoons. The events are especially characterized by their spontaneity. The service usually starts with a short speech that can also be given by one of the regular visitors. Songs are sung in German, Spanish, or Portuguese, followed by a sermon. The style of preaching has an anecdotal character and contains stories about experiences in Germany or biographical memories. In addition to the sermons, there are always opportunities for personal testimonies.

Spiritual warfare plays a recurring role for the community. Demons are blamed for injustice, marginalization, and sexual aberrations. Because of the manageable size of the church, almost every parishioner has a supporting role during the service, be it in the band, in the distribution of communion, offering the sermon, giving announcements, or in the preparation of the common meal after the service. Especially the dinner after the church service is the place where congregants have social interactions and exchange ideas on a wide variety of topics. Children run around and play in the worship room until the adults finally clean up and say goodbye to each other. Although the church is a relatively small community and has only few financial resources, the community is highly active in its social and charitable activities. During the week, the community organizes a mother-and-child café, women’s groups, or bazaars. A special highlight during the observations was a workshop on integration*.*

The purpose of the workshop was to integrate immigrant Brazilian women into the formal or informal labor market. Various exhibitors had the opportunity to offer their products and to network with each other. A Brazilian-born female lawyer who works in Germany gave a voluntary presentation on integration and specific laws which regulate the labor market or provide social welfare. Moreover, another Brazilian-born woman from the German state employment agency offered individual counseling in Portuguese language. A total of 61 people, mainly women, attended the workshop in the church.

For the *Laginha* church, it was a great success as new people came to the church to find out about it. The pastor opened the workshop by saying that it was important for the church to fulfill a social function for its fellow citizens in the city. The church and the participants of the workshop were enthusiastic about the idea of another workshop, which was originally planned for April 2020 but had to be postponed due to the COVID-19 pandemic.

On an individual level, some further insights into the church were also gained. In the interviews, two members of the *Lagoinha* church in Berlin who immigrated to Germany for different motives were interviewed. The first interview was conducted with Victoria, who is working as an entrepreneur and runs a beauty salon in Berlin. She grew up in the South of Brazil and her family belonged to Jehovah’s Witnesses. After Victoria became pregnant at a young age and as an unmarried woman, she broke with her religious community and her family. She was left on her own at an early age and became a member of a Pentecostal church in Brazil. After several years, Victoria left Brazil and moved to Portugal, where she met a German boyfriend, whom she followed to Berlin while her daughter studied in Australia. Especially moments of solitude are described by Victoria as special encounters with God, in which she gets to know God best. Victoria said that personal prayer was her most important strategy to withstand the loneliness of being separated from her friends and family members. Victoria adopted the Pentecostal practice of Bible reading in a more individual and autonomous way. Moments of loneliness became reinterpreted and took on spiritual meaning for her. This narrative pervades Victoria’s interpretations. In this respect, she did not initially need a church to continue practicing her individual faith and for the first couple of years in Berlin she did not attend any church. It was more by chance that Victoria came to know and join the *Lagoinha* church in Berlin. Belonging to the church and being in fellowship and solidarity with other members helped her to end the relationship with her ex-boyfriend and to become self-sufficient in a country which was new to her at this time and to tackle a language basically unknown. Victoria described the relationship with her German boyfriend as problematic; he took advantage of his position of power and did not give Victoria space to develop certain skills she would have needed to become independent in Germany. She explains herself as completely dependent on her partner, not only financially but also from a linguistic and social perspective. As she calls herself an evangelical, it was important to her to marry—something her boyfriend had promised her back in Portugal. But as soon as the couple arrived in Berlin and settled down, her boyfriend decided not to marry anymore, as Victoria explained. The social network of relationships, which the church provides, gave Victoria not only the material resources but rather also the emotional support during the period of separation. Victoria described the role of the pastor as that of a mother who guided her. The pastor accompanied Victoria and supported her in the bureaucratic processes becoming an entrepreneur in Berlin.

Another interview was conducted with João, who studies medicine and is preparing for his exams in Berlin to obtain a medical license. João had already lived in Moscow for several years, where he helped his brother who worked as a missionary for a Brazilian Pentecostal church. During that time, João studied medicine at the local university in Moscow and finished the curse successfully. Another brother of João, also a doctor by education, had hoped for better working and living conditions than in Russia or Brazil and had thus immigrated to Berlin. He invited João to follow his lead. Although João was uncertain about the idea to learn a new language and get acquainted with a new culture, he decided to go with his brother and sister-in-law to Berlin. However, João’s brother left Berlin only after a few months when he received an invitation to work in the south of Germany as a medical doctor. João, who had started a language course and begun his doctorate in medicine at a prestigious university hospital in Berlin, did not want to waste these opportunities but was not able to afford housing on his own. In the interview, João talked about the support he receives by being a part of the church and the global Christian community. In the *Lagoinha* church, João met an elderly Brazilian woman who has already lived in Germany for a long time and was married to a German man. Her children had moved out of the house recently, and she and her husband had room for another person. She did not know the young student well, but since he had revealed himself as an evangelical, she felt he was trustworthy and offered him to stay in her house for as long as needed. João used this opportunity. In his narrative, he combines this memory with the theological explanation that God would always provide for him through others. João also received support in overcoming bureaucratic hurdles, in which the pastors helped him to fill in the documents he needed for the university in Berlin or his visa documents. The interviewee referred to his pastors as his spiritual parents and the other members in the church as his brothers and sisters because they did support him in so many ways and relate to him through their common faith. The practical benefits for individual immigrants are obvious. The *Lagoinha* strives and is committed to address and connect different migrants in and outside the community with each other. The pastors are not primarily concerned with addressing an exclusively Brazilian-born clientele through services and do not define the church as an exclusively Brazilian congregation. The described functions were also particularly visible in the individual interviews. The interpretation and reinterpretation of the patterns of explanation of the respective theology and worldview obviously change the realities of the interviewees in a powerful way. In Victoria’s case, loneliness no longer becomes a threat, but a communion with God. João benefits from other people no longer seeing him as a stranger but as a trustworthy person through his belonging and eventually becoming a literal family substitute for him.

### Igreja Universal do Reino de Deus

The *Igreja Universal do Reino de Deus (IURD)* is a prominent example of the global spread of Brazilian neo-Pentecostalism and has been the subject of many international academic contributions (Freston 2005, van Wyk 2015, de Castro Moreira 2020, Openshaw 2020, 2021). Founded in 1977, the church spread rapidly to various cities and states in Brazil and expanded quickly to various other countries.

According to the register of associations in Berlin, the German branch of the *IURD* was founded in 1995 in Frankfurt as a registered association.^6^ However, the church activities seem to have started earlier. According to Rodrigues and Silva (2014), who mentioned the *IURD* in Berlin in one of their articles, the congregation started its activities as a small group back in the early 1990s.

The national network consists of 11 congregations in different German and Austrian cities and is rather small compared to the networks in other European countries such as Spain, France, or the UK. The *IURD* is constantly proclaiming new church plants via social media. The church rents conference rooms in hotels in different cities in order to hold church services to show its presence in those cities. While the head pastor of the *IURD* in Germany originally is from Portugal, many of the other pastors were born and raised in Brazil.

Since its existence in Germany, the church has changed its name several times. While Freston (2001) observed that the church in South Africa changes its name frequently in order to avoid legal issues due to public scandals, in Germany, however, the congregation seems to change its name in particular for linguistic reasons. In 1995, the official name was *Kirche Universal Reich Gottes e. V.*,^7^ which is a literal translation of the Brazilian name *Igreja Universal do Reino de Deus* but is grammatically incorrect in German. Gradually, the church made linguistic adjustments and between 2000, its official name was *Universal Kirche vom Reich Gottes e.V.*.^8^ In 2003 however, the church changed its name again and since then *Hilfszentrum UKRG e.V.* (engl. Help Centre UCKG) has been its official name.

Since 2016, the *IURD* is located in Wedding, one of the most culturally, religiously, and linguistically diverse districts in Berlin, located only a few kilometers away from the *Reichstag* and the new government district, where it has recently bought a traditional neo-Gothic edifice. The purchase of a representative building in Berlin has given nationwide media attention to the church for the first time. Apart from media coverage of the election success of Jair Bolsonaro in 2018 and the purchase of church buildings in Stuttgart (2017) and Berlin (2019), the church in Germany themselves have received little to no media attention so far. In contrast to some Lusophony countries, in which the *IRUD* has grown enormously and caused severe scandals, the church in Germany remains a small congregation and does not attract a lot of attention at all.

The *IURD* lists, according to its own accounts, several hundred members in Germany.^9^ During the fieldwork, I had several encounters with adherents who can be described as dedicated and eager in acquiring new adherents and following the pastor’s guidelines. Many of the congregants attend the various church services and events several times per week.

Regarding the demographic characteristics of the audience of the *IURD*, it is important to highlight two crucial points.

First, although many adherents are female immigrants from Brazil, the church audience also includes immigrants especially from eastern Europe and other non-Lusophony countries.

Second, alongside immigrants, the *IURD* also attracts comparatively many people born Germany and having no immigration background at all. The church has become a central aspect of their lives; some of them met a Brazilian partner through the community. They made pilgrimages to the inauguration of the *Temple of Solomon* in São Paulo and perceive Brazil as a new center of a global Christian awakening. Many of them struggled financially earlier in their life or suffered from psychological problems, such as depression, and they have found a connection to the theologies, spiritual approaches, and practices of the church, as they repeatedly confessed in their testimonies during the services or in the informal talks.

During my research in the short period of time, I was not able to conduct any interview among the congregants of the *IURD*. Even though the pastors of the congregation were open to my research and supportive of it—I was allowed to move freely in the congregation, attend services, and interview participants—the congregants were skeptical about participating in academic research. This experience does match other experiences from previous research projects in other *IURD* communities in other countries, where worshipers were skeptical of people with a media or scientific interest. (Van Wyk 2015; Openshaw 2020).

However, during the participant observations, the personal testimonies of the members provided some explicit and valuable information about the religious perceptions inside the church. A young woman from Angola reported in one of the testimonies during the church service that she was threatened with termination of employment immediately before her visa was about to expire. To renew her visa, she needed an employment contract that assured that she would receive enough income to make a living in Germany on her own. For the extension of her work contract, according to her testimony, the woman prayed intensively, participated in prayer campaigns, and faithfully gave tithes and additional donations, through which she wanted to receive God’s support. Finally, she got the message that her work contract would be extended. The woman attributed the approval of the employer and the immigration office to the intervention of God, who answered her prayers for giving the money and faithfully participating in the church’s programs. The ecclesiastical institution and its pastors are recognized by the faithful with high authority and much power, and their instructions enable direct communication with the divine. Unlike the *Videira*, however, it is not social fellowship and mutual prayer that people experience as supportive in the problems of everyday life, but proximity to the altar, exorcisms, donations, and the repetition of the pastor’s prayer formulas, as the observations showed. In this sense, the church is understood as a strong mediating authority, which represents an immediate connection to the spiritual world and can intervene in certain needs abroad, such as the issuing of visa documents.

The *IURD* is usually characterized by its top-down government, its systemized rituals across time and space, and uniformed appearance and routines (Oosterbaan 2017). Even abroad, the church distinguishes itself by its specific practices and theologies and rarely combines them with local adaptations. Structures, programs, and prayer chains used in Brazil, Australia, Sweden, and other countries are also applied in Germany. Thus, three to four events per day are offered, although attendance tends to be low. The church attracts not only Portuguese-speaking migrants but people who are at the margins of society, have experienced great frustrations and diseases, and are confident that God will intervene in their lives to bring about financial or social justice.

The relationship between the German host society and the *IURD* can certainly be described as difficult. In the news magazines of the secular media as well as in publications of the Protestant and Catholic mainline churches, the *IURD* is treated critically, especially regarding their wealth and political power in Brazil. Which specific functions the *IURD* could fulfill remains an open question. People reinterpret their situation and may find help and support through the practices of the church. In order to answer the question properly, however, further research about the *IURD* in Germany would be a worthwhile project.

## Conclusion

The four Brazilian churches in Berlin that were considered in this article differ from one another in various ways and a direct comparison remains difficult. Brazilian migration to Germany can be understood as a multiplier of religious pluralism that remains largely unexplored. The *Igreja Universal do Reino de Deus* and the *Assembléia de Deus*—both prominent in the literature of global Brazilian Pentecostalism—continue to display marked differences and demarcations that remain effective in Berlin. The results indicate that it is not only a function of building a bridge between the Brazilian context of origin and the German host society that the churches must perform, but the social realities of the congregations and its members reveal even more complexity.

A first, complexity regards the presentation of the churches as exclusively»Brazilian« or»Lusophony« communities: The *Igreja Universal do Reino de Deus* does not only draw migrants from other Lusophony countries, such as Portugal, Angola, and Mozambique, but also welcomes other migrant or non-migrant groups; the *Assembléia de Deus*, on the other hand, is a congregation dominated by Brazilian migrants, where, in addition to religion, national identity is reaffirmed. By meeting other Brazilians of the diaspora at local Pentecostal churches in Berlin and through discussions about popular Brazilian television shows, such as soap operas and TV programs, by sharing cooking recipes adapted to local ingredients, or by exchanging addresses of Brazilian groceries, the congregants stress their national coherence and reaffirm a national identity in the diaspora. However, a perspective that uses only nationalities, languages, or culture as characteristics to describe the observed churches in Berlin can obscure the intercultural and international diversity inside the different churches. In this sense, social interaction in the churches itself can already provoke processes of interaction and negotiation, promoting the identification of differences and commonalities and eventually leading to individualization.

Paradoxically, striking similarities can be observed among the Brazilian churches in Berlin, which cannot be explained easily. None of the Brazilian Pentecostal churches in Berlin has integrated itself into the German Pentecostal association *(BfP*) so far, which would mean valuable support for them and help solving bureaucratic issues*.* As Währisch-Oblau (2006) has already noted regarding African migrant churches in Germany, the Pentecostal churches react suspiciously to the *BfP*, as the structural integration into a German Pentecostal umbrella organization runs counter to the churches’ strong sense of reverse-mission. Whether this analysis also applies to Brazilian Pentecostal churches has yet to be investigated The *Igreja Universal do Reino de Deus*, *Videira*, and *Lahoinha* are active in missionary and reaching out to attract others.

The functions the churches offer to their members, however, are markedly different and they cannot be applied in a generalizing way, as the described functions of *da Silva Moreira* might suggest. Some of the interviews reveal even inconsistencies regarding the function of traditional belongings which might be disembedded by Pentecostalism. During the process of migration and integration into an unfamiliar society, migrants are often automatically forced to use a new language, to acquire new habits, and to build new friendships, which leads to the weakening of traditional belongings and can therefore not only be regarded to the affiliation to Pentecostalism.

On the other hand, the reinterpretation of traditional interpretive patterns can create new problems for migrants if they engage in using their faith to solve visa problems, for example. Religious organizations might not directly influence governmental and bureaucratic hurdles, but they might be interpreted as doing so. As became apparent during my fieldwork, social realities and traditional interpretations determine the topics in many of the churches and influence ideas and imaginations of God.

The diversity of the Lusophony Pentecostal churches in Berlin reflects the diversity and fractions of their contexts of origin. Demarcations between Pentecostal and neo-Pentecostal churches remain visible and effective, also in Berlin.

However, to distinguish between Pentecostal and Charismatic churches is sometimes ambiguous. Most of the interviewees did not want to be categorized as Pentecostals, neo-Pentecostals, or Evangelicals but simply be called and understood as Christians. This can be interpreted as an expression of the congregants’ self-concept of being understood not as members of an exclusive Brazilian religious community, but as part of an international group, which could strengthen da Silva Moreria’s described function about the global identity of Pentecostal parishioners.

As researchers from the new expanding field of Pentecostal studies have pointed out, Pentecostalism contains a huge diversity. Its ideas and practices are only held together by loose threads, and churches differ greatly regarding their organizational structures and socio-economic backgrounds (Haustein et al. 2015). The Lusophony Pentecostal landscape in Berlin made this diversity very visible. The role of Pentecostal churches regarding the research question can therefore only be described as ambiguous. Some of the examples demonstrate the equivocal nature of religion in modern societies. Local contexts can change, and social status can be reinterpreted under the lens of a Pentecostal interpretation, such as in the case of Anna and Victoria. However, belonging to a Pentecostal congregation can also be understood in an exclusive sense, in which social cohesion circulates almost solely on memories and shared knowledge among a mono-ethnic and mono-religious group.

## Footnotes

1 https://www.bfp.de/statistiken-und-zahlen-zum-bfp (last accessed 30/09/2021).

2 http://www.migrationskirchen-in-berlin.de/index.php/migrationsgskirchen/184-migrationskirchen-in-berlin (last accessed 30/08/2021).

3 https://www.gov.br/mre/pt-br/search?SearchableText=brasileiros%20no%20mundo (last accessed 30/08/2021).

4 http://igrejasnaalemanha.blogspot.com/ (last accessed 28/09/2021).

5 https://lagoinha.com/lagoinha-news/19683/serie-conheca-a-lagoinha-mais-perto-de-voce (last accessed: 30/08/2021).

6 District Court File: Amtsgericht Charlottenburg. File: Hilfszentrum UKRG e.V. Geschäftsnummer: VR 2001B. Gründungsversammlung der „Kirche Universal Reich Gottes e.V.“ 30.10.1995.

7 District Court File: Amtsgericht Charlottenburg. File: Hilfszentrum UKRG e.V. Geschäftsnummer: VR 2001B. Gründungsversammlung der „Kirche Universal Reich Gottes e.V.“ 30.10.1995.

8 District Court File: Amtsgericht Charlottenburg. File: Hilfszentrum UKRG e.V. Geschäftsnummer: VR 2001B.

9 https://hilfszentrum.de/die-institution/ (last accessed 30/08/2021).
